# A Selective Irreversible Inhibitor of Furin Does Not Prevent *Pseudomonas Aeruginosa* Exotoxin A-Induced Airway Epithelial Cytotoxicity

**DOI:** 10.1371/journal.pone.0159868

**Published:** 2016-07-26

**Authors:** Timothy E. G. Ferguson, James A. Reihill, Brian Walker, Robert A. Hamilton, S. Lorraine Martin

**Affiliations:** Biomolecular Sciences Research Group, School of Pharmacy, Queen’s University Belfast, Northern Ireland, United Kingdom; University of Gdansk, POLAND

## Abstract

Many bacterial and viral pathogens (or their toxins), including *Pseudomonas aeruginosa* exotoxin A, require processing by host pro-protein convertases such as furin to cause disease. We report the development of a novel irreversible inhibitor of furin (QUB-F1) consisting of a diphenyl phosphonate electrophilic warhead coupled with a substrate-like peptide (RVKR), that also includes a biotin tag, to facilitate activity-based profiling/visualisation. QUB-F1 displays greater selectivity for furin, in comparison to a widely used exemplar compound (furin I) which has a chloromethylketone warhead coupled to RVKR, when tested against the serine trypsin-like proteases (trypsin, prostasin and matriptase), factor Xa and the cysteine protease cathepsin B. We demonstrate QUB-F1 does not prevent *P*. *aeruginosa* exotoxin A-induced airway epithelial cell toxicity; in contrast to furin I, despite inhibiting cell surface furin-like activity to a similar degree. This finding indicates additional proteases, which are sensitive to the more broad-spectrum furin I compound, may be involved in this process.

## Introduction

Furin is a type I transmembrane serine protease ubiquitously expressed in vertebrates that cycles from the trans-Golgi network through the endosomal system to the cell membrane and back [1]. Furin catalyses the hydrolysis of precursor peptide and protein substrates including receptors, hormones and cell surface proteins [2]. Numerous reports demonstrate that furin contributes to the pathology of a variety of diseases via the activation of several viral and bacterial pathogenic proteins, including *P*. *aeruginosa* exotoxin A (PEA) [3,4]. Chronic infection of the airways by *P*. *aeruginosa* is a central feature of cystic fibrosis (CF) lung disease [5], with PEA detected in the respiratory secretions of patients with CF [6]. Antibodies to PEA have also been identified in the sera of CF patients suffering from chronic *P*. *aeruginosa* infection [7]. The mortality rate among patients infected with PEA-producing *P*. *aeruginosa* isolates has been reported as 3 times higher compared with infected non-producers [8]. Furin is also implicated in tumorigenesis, atherosclerosis, diabetes and neurodegenerative disorders such as Alzheimer’s disease [3,9,10]. Novel furin inhibitors are therefore of prime importance as research tools and potential therapeutic agents.

A number of synthetic furin inhibitors have been developed over recent decades including the widely used compound furin I (Decanoyl-RVKR-chloromethylketone (CMK)). Furin I contains a specificity element (RVKR) based on furin substrate specificity for the consensus amino acid sequence Arg-X-Lys/Arg-Arg (where X is any amino acid and indicates the point of cleavage).

Peptidyl chloromethyl ketones (CMK) were originally identified as potent inhibitors of serine proteases [11,12] forming irreversible transition state analogues through rapid alkylation of the active-site histidine residue. These compounds have been employed widely for obtaining valuable crystallographic information on the structure of protease active-sites and thus highlighting their proteolytic mechanisms. Unfortunately, a number of drawbacks are associated with the use of CMK-based inhibitors, which limits their potential value and application. The highly electrophilic CMK warhead has subsequently been shown to display poor selectivity for serine proteases resulting in alkylation of both cysteine proteases including cathepsin B [11,13] and the active-site threonine residue of the proteasome [14]. Furthermore bionucleophiles such as glutathione are also rapidly alkylated by the CMK electrophile [15]. In addition, CMKs also possess poor aqueous stability [15] as a consequence of the highly reactive nature of the warhead, limiting their utilisation in biological studies.

The application of biotinylated active site-directed inhibitors or affinity labels was pioneered within our group as a method to allow for the disclosing and profiling of cysteine and serine proteolytic activities in various biological media. These affinity labels allow for the detection of proteases containing the active-site tethered biotinylated affinity labels following disclosure using streptavidin-based systems. This work was initially carried out with biotinylated peptidyl diazomethane probes for cathepsin B-like proteases [16] and was also shown to be applicable with the use of peptidyl CMK probes [17]. Similarly, we were also the first group to synthesise biotinylated peptidyl diphenyl phosphonate compounds that specifically inhibit serine proteases and display less limitations than peptidyl CMKs. These were subsequently applied to the selective disclosure and detection of serine proteases produced by breast cancer cells [18] and the digenetic trematode *Haplometra cylindracea* [19]. Herein we report the development of a novel compound *N*^*α*^-Pal-*N*^*ω*^-(Biotinyl-Ahx)-Lys-RVKR^P^(OPh)_2_ (QUB-F1) consisting of a furin specificity element (RVKR) and a diphenyl phosphonate electrophilic warhead. We hypothesised that QUB-F1 would exhibit a greater degree of selectivity than the highly reactive furin I. The incorporation of a biotin moiety also facilitates visualisation and activity profiling of furin-like proteases. As a more selective inhibitor of furin, QUB-F1 was subsequently used to investigate the role of furin in the activation of PEA.

## Materials and Methods

### Materials

Recombinant human enzymes were sourced as follows: furin and matriptase (R&D Systems); prostasin (Sino Biological); trypsin and chymotrypsin (Sigma-Aldrich); factor Xa and neutrophil elastase (Merck Millipore) and proteinase-3 and cathepsin G (Elastin Products, Missouri). Fluorogenic substrates were sourced as follows: *p*-Glu-Arg-Thr-Lys-Arg-NH_2_Mec and Boc-Gln-Ala-Arg-NH_2_Mec (R&D Systems); Boc-Ile-Glu-Gly-Arg-NH_2_Mec (Bachem); Z-Gly-Gly-Arg-NH_2_Mec, MeOSuc-Ala-Ala-Pro-Val-NH_2_Mec and Suc-Ala-Ala-Pro-Phe-NH_2_Mec (Sigma-Aldrich). All chemicals and reagents for the synthesis of QUB-F1 were supplied by Sigma-Aldrich unless otherwise indicated. Fmoc-Lys(Boc)-SASRIN resin was sourced from Bachem. Fmoc-protected amino acids and HCTU were supplied by Merck Millipore.

### QUB-F1 synthesis

QUB-F1 (S1 Fig) was synthesised using a combination of solid and solution phase methodologies. *H*-*N*^*ω*^,*N*^*ω’*^*-*(di-Boc)-Arg^P^(OPh)_2_ was synthesised as described previously [20]. The protected peptide *N*^*α*^-Pal-*N*^*ω*^-(Biotinyl-Ahx)-Lys-*N*^*ω*^,*N*^*ω’*^-(di-Boc)-Arg-Val-*N*^*ω*^-(Boc)-Lys-*OH* was synthesised on a Fmoc-Lys(Boc)-SASRIN resin using standard Fmoc/*t*-Butyl methodologies, followed by cleavage with 1% (v/v) trifluoroacetic acid/dichloromethane. The protected peptide acid was subsequently coupled to *H*-*N*^*ω*^,*N*^*ω’*^*-*(di-Boc)-Arg^P^(OPh)_2_ in a HCTU-mediated condensation reaction and final deprotection was carried out with trifluoroacetic acid. Confirmation of the correct compound was achieved through HPLC and mass spectroscopy. ESI-MS *m/z* 363.15 [M + 4H^+^]/4 (calculated C_72_H_123_N_16_O_11_PS: 1451.91).

### Determination of second order rate constant (QUB-F1 versus recombinant human furin)

A range of final inhibitor concentrations (at least 5) were prepared from a 10 mM stock solution of QUB-F1 (in *N*,*N*-dimethyformamide (DMF)). The fluorogenic substrate, *p*-Glu-Arg-Thr-Lys-Arg-NH_2_Mec (pERTKR-AMC), was diluted in assay buffer (25 mM Tris/HCl containing 1 mM CaCl_2_, pH 7.4) and used at a fixed concentration of 50 μM throughout. All inhibition assays were performed in microtitre plates maintained at 37°C in a final volume of 100 μl. The reaction was initiated by the addition of recombinant human furin (0.01 μg/well) and the rate of substrate hydrolysis continuously recorded at λ_ex_ 360 nm, λ_em_ 480 nm, over a period of 90 minutes using a FLUOstar Optima microplate reader (BMG Labtech). The resultant inhibition progress curve for QUB-F1 was then analysed according to the kinetic models developed by Tian and Tsou [21] and Walker and Elmore [22], for irreversible inhibitors, using GRAFIT (Erithacus Software). The inhibitor constant K_i_, the apparent first order rate constant k_3_, and over all second-order rate constant (k_3_/K_i_) for QUB-F1 against furin was determined, from a plot of 1/A against [I] (where A is the apparent second-order rate constant for irreversible inhibition determined for each inhibitor concentration [I]). The determination of K_m_ and V_max_ were also determined separately by fitting initial rates of hydrolysis to substrate concentration, according to Michaelis-Menten kinetics, also using GRAFIT (Erithacus Software). The inhibitor constant K_i_, the apparent first order rate constant k_3_, and the overall second-order rate constant (k_3_/K_i_) for QUB-F1 against furin were determined, from a plot of 1/A against [I] (where A is the apparent second-order rate constant for irreversible inhibition determined for each inhibitor concentration [I]).

### Fluorogenic protease activity assays

Recombinant protease activities were assessed using the cognate fluorogenic substrates (final concentration 50 μM) as follows: trypsin (Z-Gly-Gly-Arg-NH_2_Mec), prostasin and matriptase (Boc-Gln-Ala-Arg-NH_2_Mec), factor Xa (Boc-Ile-Glu-Gly-Arg-NH_2_Mec), neutrophil elastase and proteinase 3 (MeO-Suc-Ala-Ala-Pro-Val-NH_2_Mec), chymotrypsin and cathepsin G (Suc-Ala-Ala-Pro-Phe-NH_2_Mec). The rate of substrate hydrolysis in the presence or absence of furin inhibitors was assessed by continuous recording at λ_ex_ 360 nm, λ_em_ 480 nm, over a period of 60 minutes using a FLUOstar Optima microplate reader (BMG Labtech).

### Western blotting analysis

Recombinant human furin (0.4 μg) was incubated with QUB-F1 (100 μM) for 45 minutes at 37°C. The sample was then denatured and reduced in Laemmli treatment buffer for 5 minutes at 95°C, resolved by SDS-PAGE, before transfer onto a nitrocellulose membrane which was blocked with a solution of Tris-buffered saline (TBS) containing 3% (w/v) bovine serum albumin and 0.1% (v/v) Tween-20. The membrane was subsequently probed with a streptavidin-Horseradish peroxidase conjugate (1:10,000) (Vector-Laboratories) in blocking solution for 1 h prior to detection by chemiluminescence (Luminata Forte Western HRP substrate (Millipore)).

### Cell culture

The CuFi-1 cell line was derived from human airway epithelium of a CF patient (F508del/F508del) transformed with a reverse transcriptase component of telomerase, hTERT and human papillomavirus type 16 E6 and E7 genes [23]. Cells were grown as submerged cultures using bronchial epithelial growth medium (BEBM including a supplement pack) containing: epidermal growth factor, hydrocortisone, bovine pituitary extract, transferrin, bovine insulin, triiodothyronine, epinephrine, retinoic acid, penicillin-streptomycin (0.025 μg/ml), gentamycin (0.05 ng/ml) and amphotericin (25 μg/ml).

### Cytotoxicity assay

Cytotoxicity was determined using the LDH-Cytotoxicity Colorimetric Assay Kit II (Bio Vision) in accordance with the instructions provided by the manufacturer. Addition of the supplied cell lysis solution to control wells was used to determine 100% cytotoxicity in each set of experiments.

### Epithelial cell surface furin-like activity assay

Cell culture media was removed from CuFi-1 epithelial cells grown in 96 well microtitre plates (Thermo Scientific Sterilin; clear bottom, black walled) and replaced with PBS /+ inhibitor compounds (furin I or QUB-F1). The furin fluorogenic substrate (pERTKR -NH_2_Mec) was then applied directly to the cell cultures to give a final concentration of 50 μM. Cell surface proteolytic activity was measured by monitoring the formation of–NH_2_Mec at λ_ex_ 360 nm and λ_em_ 480 nm using a 5 mm orbital scan every 60 s over a 1 h period (FLUOstar Optima microplate reader; BMG Labtech).

### Statistics

Results are expressed as the means ± s.e.m. with statistically significant differences determined using a two-tailed Student’s t test, with P<0.05 deemed significant.

## Results and Discussion

**Fig 1:**
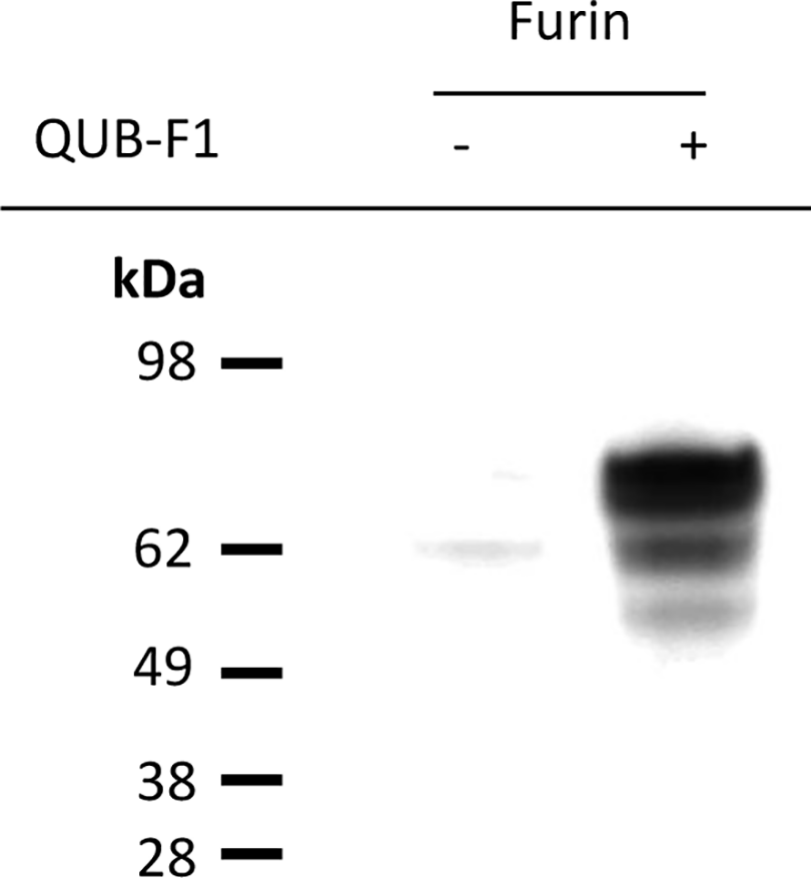
Irreversible inhibition and visualization of recombinant human furin by QUB-F1. Western blot analysis was performed under reducing and denaturing conditions demonstrating QUB-F1 (100 μM) is irreversibly bound to furin (0.4 μg). The inhibitor-proteases complex can be visualized using streptavidin-HRP, which detects the biotin reporter group on the compound.

The ability of QUB-F1 to inhibit recombinant human furin was assessed using a steady-state fluorimetric assay employing the cognate fluorescent substrate (pERTKR-NH_2_Mec). The k_3_, K_i_ and k_3_/K_i_ values where determined as follows: K_i_ 9.33 (± 0.32) x 10^−7^ M, k_3_ 0.016 (± 0.004) min^-1^ and k_3_/K_i_ 2.05 (± 0.112) x 10^4^ M^-1^ min^-1^. This is an increase in potency in comparison to the previously reported single amino-acid broad-spectrum inhibitor QUB-TL1 [20]. The improved inhibitory potency can be attributed to the inclusion of a furin specificity sequence (RVKR) within QUB-F1 providing increased affinity for the furin active site. The kinetic data is also consistent with QUB-F1 acting as an irreversible inhibitor, forming an irreversible covalent complex with the furin active-site. This was confirmed by Western blotting where recombinant human furin inactivated by QUB-F1 and denatured under reducing conditions allowed successful visualization by streptavidin-HRP (which detects the biotin tag incorporated into the compound) (Fig 1). This observation highlights QUB-F1 may potentially be employed as a research tool enabling profiling of furin activity in biological samples.

**Fig 2:**
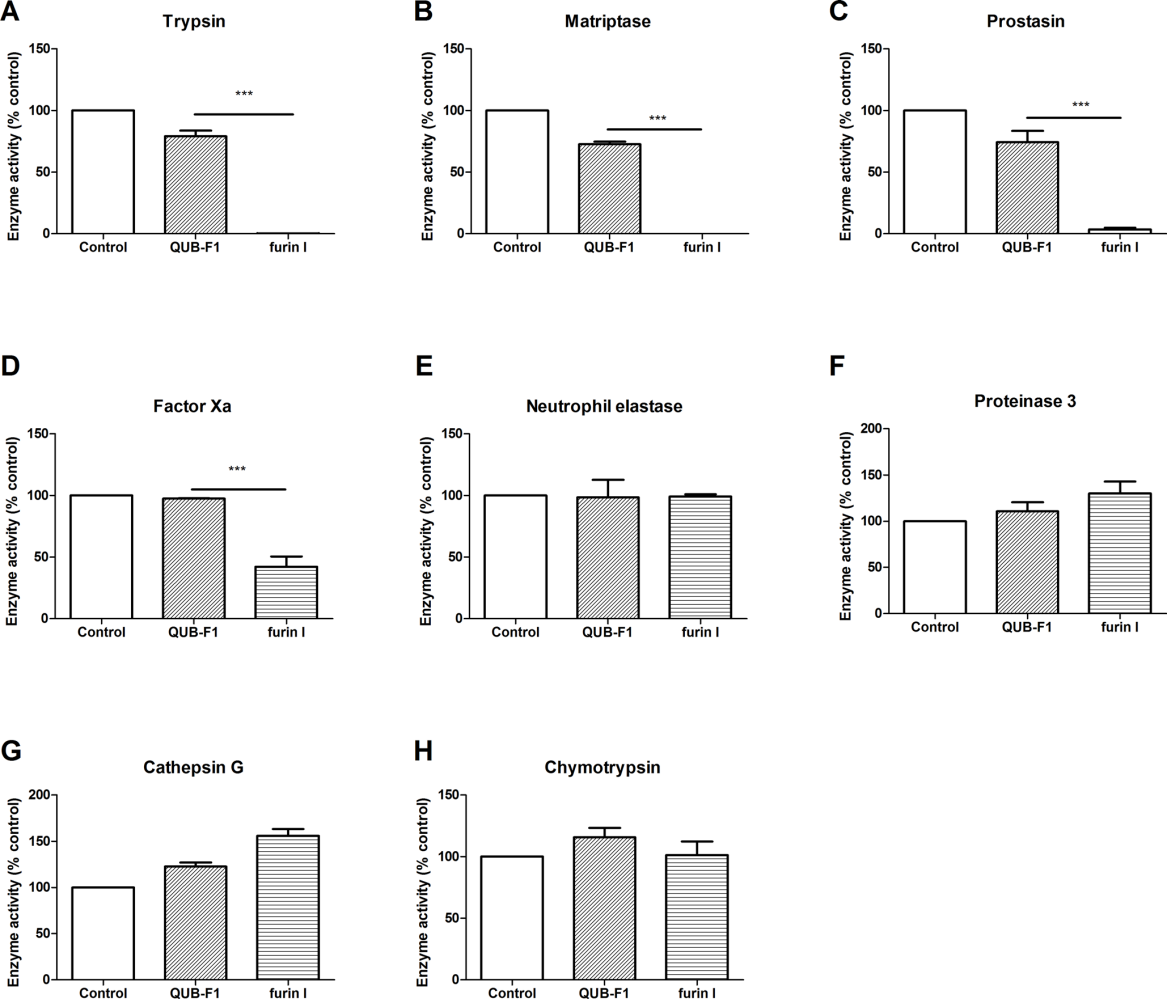
Evaluation of the selectivity of action of QUB-F1 and furin I versus a selection of serine proteases. Trypsin (A), matriptase (B), prostasin (C), factor Xa (D), neutrophil elastase (E), proteinase 3 (F), cathepsin G (G) and chymotrypsin (H) activity was assessed using their cognate fluorogenic substrates in the presence or absence of 10 μM inhibitor (QUB-F1 or furin I) (N = 3). Data represent mean ± s.e.m.; ***P<0.001.

**Fig 3:**
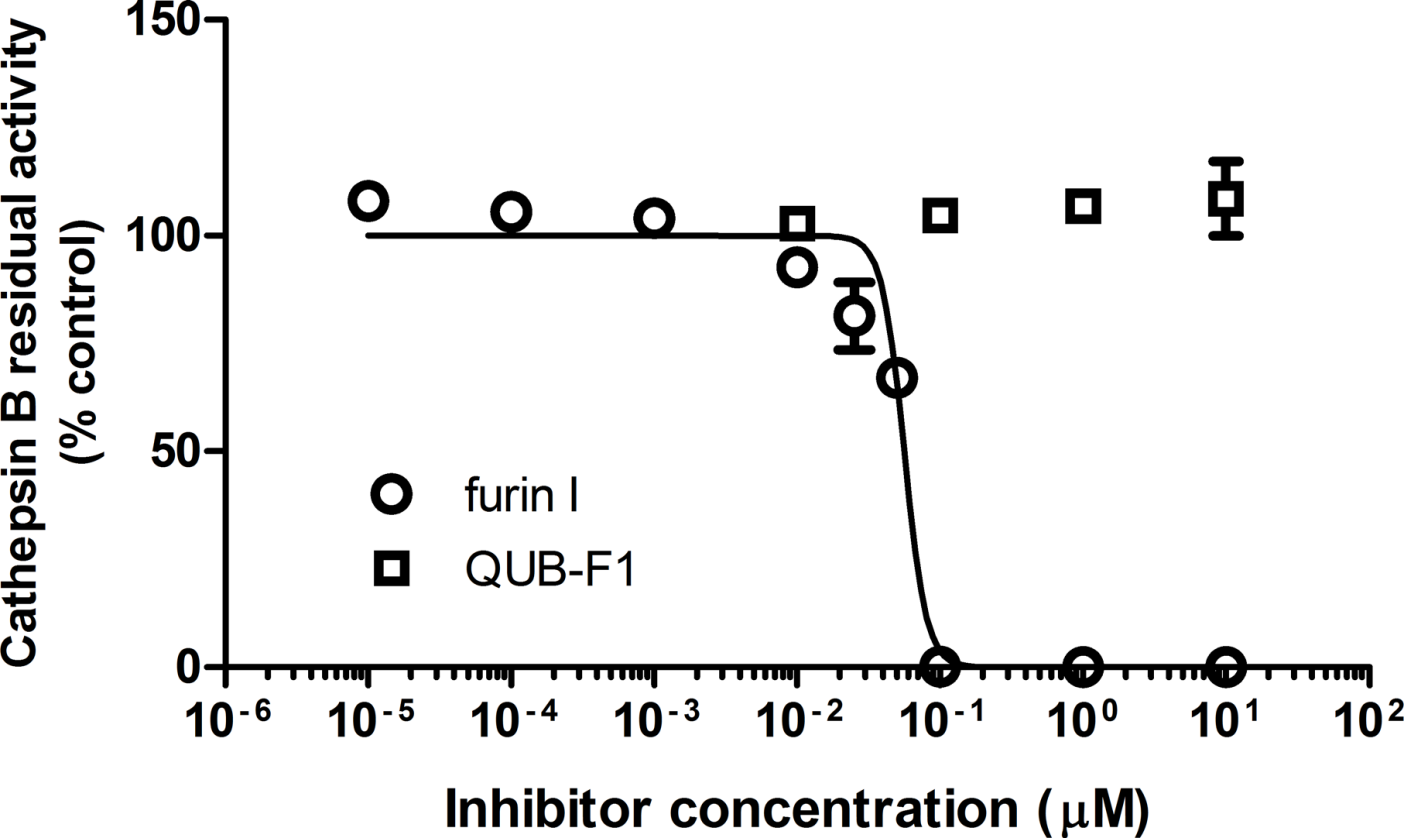
Comparison of QUB-F1 and furin I versus cathepsin B. Cathepsin B activity was assessed in the presence or absence of various concentrations of QUB-F1 or furin I (N = 3). Data represent mean ± s.e.m.

In order to assess their respective selectivity of action, QUB-F1 and furin I were tested against a selection of serine proteases (trypsin, prostasin, matriptase, factor Xa neutrophil elastase, proteinase 3, cathepsin G and chymotrypsin) relevant in CF airways. Furin I (10 μM) caused significant inhibition of trypsin-like enzymes (trypsin, prostasin and matriptase) and factor Xa; in contrast to QUB-F1 (10 μM) which did not (Fig 2A–2D), indicating improved selectivity of the latter. Neither compound was found to inhibit neutrophil elastase, proteinase 3, cathepsin G or chymotrypsin (Fig 2E–2H). Since previous work has demonstrated that peptidyl CMKs inhibit cysteine proteases irreversibly [11]; we performed a head to head comparison on the ability of furin I (an example inhibitor of this type) and QUB-F1 to inhibit the lysosomal cysteine protease cathepsin B. Furin I potently inhibits this enzyme (IC_50_ 0.056 μM), whereas QUB-F1 displayed no inhibitory action up to and including a concentration of 10 μM) (Fig 3).

**Fig 4:**
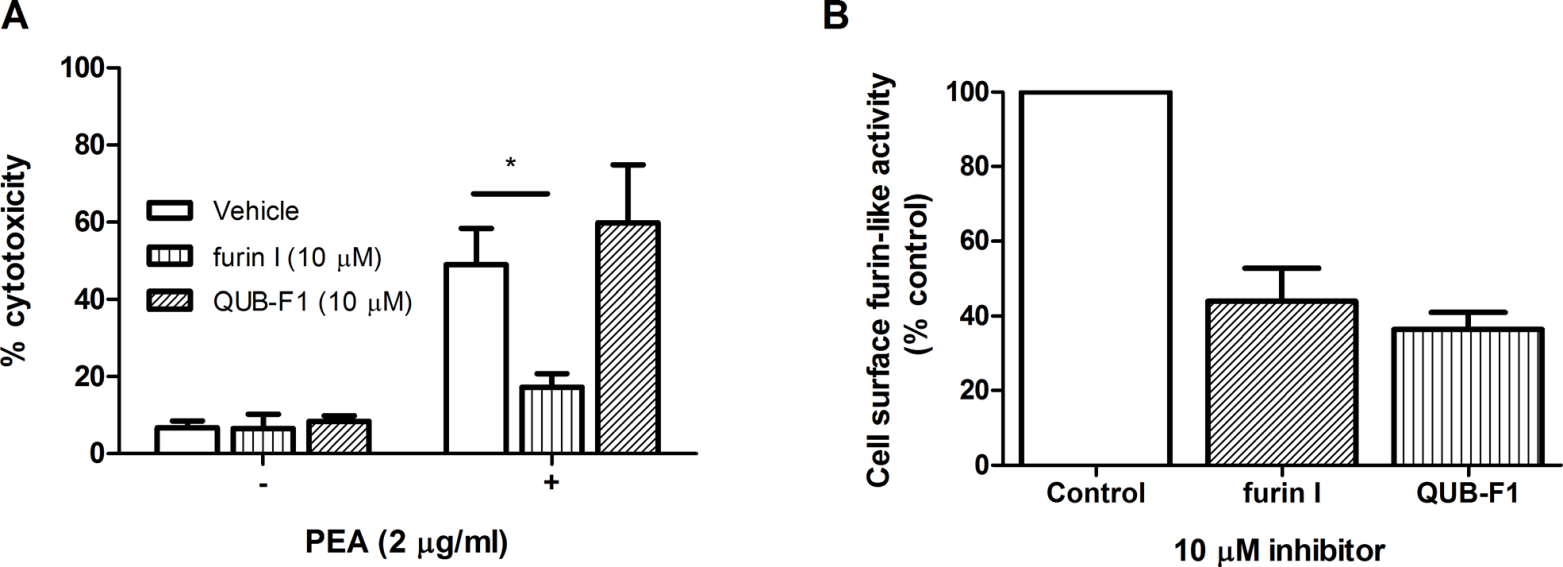
Effect of furin inhibitors on PEA-induced epithelial cell toxicity. (A) Cell toxicity (%) induced by 2 μg/ml PEA (24 h) was measured in the presence or absence of furin I or QUB-F1. Data represent mean ± s.e.m. *P<0.05. (B) Airway epithelial cell surface furin-like activity was assayed upon application of the furin fluorogenic substrate (pERTKR) to cell cultures in the presence or absence of 10 μM furin I or QUB-F1.

Furin has been implicated in the processing of PEA resulting in the generation of a 37 kDa C-terminal fragment that translocates to the cytosol causing cell death by affecting protein synthesis of host cells [24]. As furin I has previously been shown to protect CF airway epithelial cells from the toxic actions of PEA [25], we sought to determine whether QUB-F1 would have a similar impact. As expected, furin I significantly reduced PEA-induced epithelial cell toxicity in our study (Fig 4A). In contrast, however, QUB-F1 offered no protection against PEA-induced cell death (Fig 4A) even though this compound inhibited cell surface furin-like activity to the same extent as furin I (Fig 4B). Together these data suggest the involvement of proteases, in addition to furin in this process, which are not inhibited by the more specific QUB-F1 compound (Figs 2 and 3). Indeed cysteine cathepsins, which are inhibited by furin I, have previously been implicated in the PEA intoxication process [26]. The involvement of additional proteases is further indicated in experiments using furin deficient cells wherein some sensitivity to PEA is retained [27].

Future studies utilising furin-deficient and expressing cell lines, and a range of selective protease inhibitors (including for trypsin-like and cysteine proteases) may prove useful in further characterising the underlying molecular mechanisms of the PEA intoxication process; and also allow further analysis concerning the specificity of furin inhibitors including QUB-F1. Moreover whilst the current study has focused on PEA intoxication one should note several bacterial toxins including anthrax toxin, aerolysin toxin (a causative agent in many food-borne illnesses), shiga toxin and diphtheria toxin are activated by furin [3]. As such it would be of interest to examine the impact of QUB-F1 in this wider context.

In summary we have developed a novel diphenyl phosphonate-based irreversible inhibitor of furin (QUB-F1) that displays enhanced selectively when compared to the widely employed compound furin I. We believe this improved selectivity may enable improved interpretation of the role of furin in biological processes. This is exemplified by our studies evaluating the impact of QUB-F1 and furin I on PEA-induced cytotoxicity. Here we show that only the latter (less specific) compound rescues the cells indicating the potential involvement of other serine and cysteine proteases, in addition to furin in this process. We caution against conclusions obtained when non-selective inhibitors such as furin I are used within biological model systems.

## Supporting Information

S1 Fig Structure of QUB-F1 and furin I. (TIF)
