# Supplementary figures and images for: A Selective Irreversible Inhibitor of Furin Does Not Prevent Pseudomonas Aeruginosa Exotoxin A-Induced Airway Epithelial Cytotoxicity
Source: PLoS One. 2016 Jul 26;11(7):e0159868. doi: 10.1371/journal.pone.0159868 (PMC4961418; doi:10.1371/journal.pone.0159868)

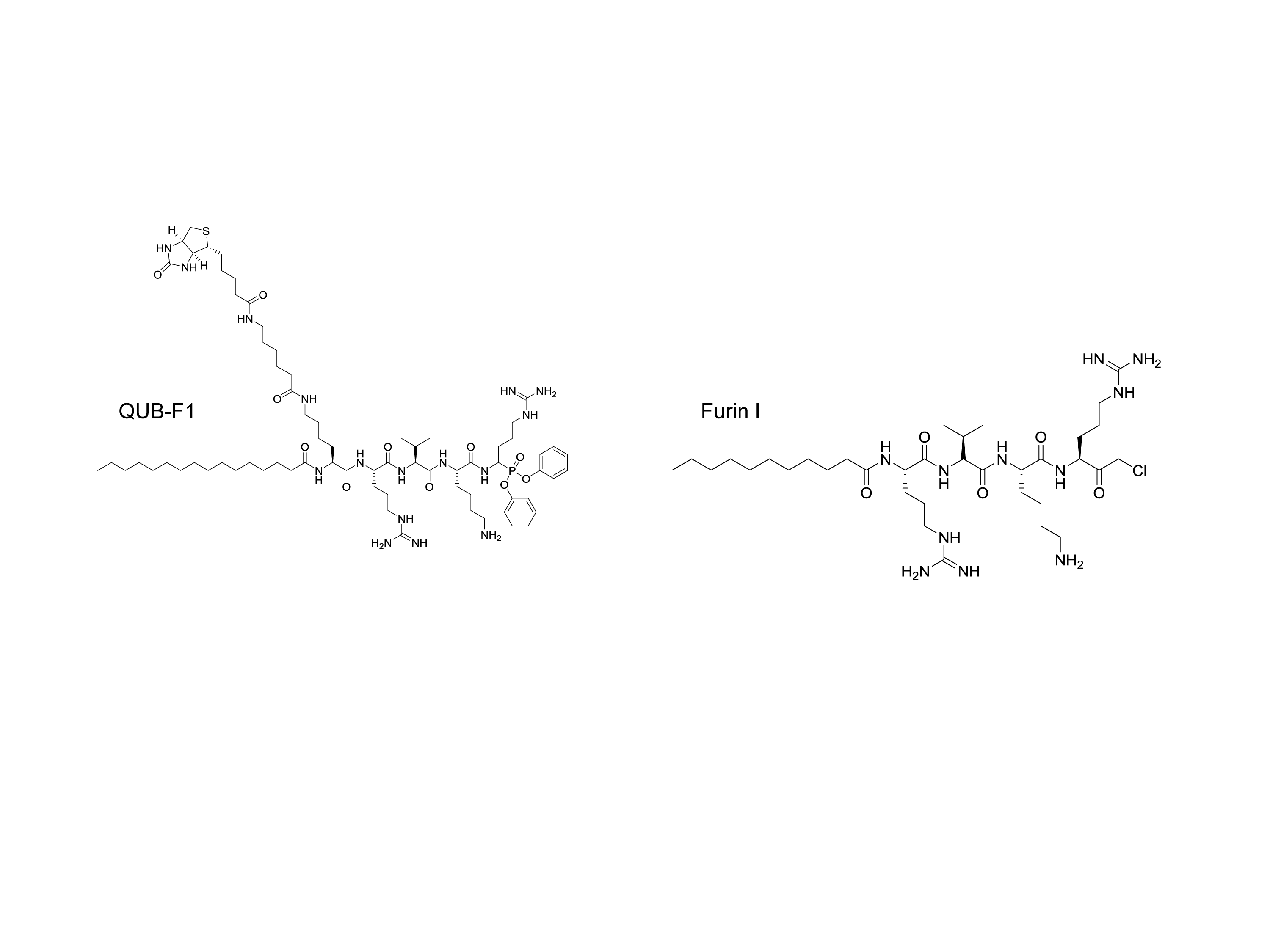

Supplement: S1 Fig — (TIF) [file pone.0159868.s001.tif]
